# The Safety of PsA-TT in Pregnancy: An Assessment Performed Within the Navrongo Health and Demographic Surveillance Site in Ghana

**DOI:** 10.1093/cid/civ625

**Published:** 2015-11-09

**Authors:** George Wak, John Williams, Abraham Oduro, Christine Maure, Patrick L. F. Zuber, Steven Black

**Affiliations:** 1 Navrongo Health Research Centre, Ghana Health Service, Navrongo, Ghana; 2 Department of Essential Medicines and Health Products, World Health Organization, Geneva, Switzerland; 3 Center for Global Health, Cincinnati Children's Hospital, Ohio

**Keywords:** group A meningococcal vaccine, PsA-TT, pregnancy, safety, Ghana

## Abstract

***Background.*** Group A meningococcal disease occurs in large epidemics within the meningitis belt of Africa that includes northern Ghana. Major epidemics in the meningitis belt have infection rates ranging from 100 to 800 per 100 000 population. In 2012, a group A meningococcal conjugate vaccine, PsA-TT (MenAfriVac), was introduced into the region in large campaigns.

***Methods.*** We report here on the safety of this vaccine when used in pregnant women in the Navrongo region of Ghana.

***Results.*** Rates of events in 1730 immunized pregnant women and their infants were compared to the rates of the same events in pregnant women who did not receive the vaccine during the campaign and also to women who were pregnant in the prior year.

***Conclusions.*** We found no evidence of any safety concerns when this vaccine was administered during pregnancy.

Group A meningococcal disease occurs in large epidemics within the “meningitis belt” of Africa, which includes northern Ghana. Major epidemics in the meningitis belt have infection rates ranging from 100 to 800 per 100 000 population [1]. These outbreaks also cause significant morbidity and mortality in pregnant women. A monovalent meningococcal group A conjugate vaccine, PsA-TT (MenAfriVac), was specifically developed to provide protection against these life-threatening epidemics [2]. There is no evidence of risk from vaccinating pregnant women or those who are breastfeeding, although live viral vaccines are generally not given during pregnancy [3, 4]. Although it is recommended that women of childbearing age be vaccinated because pregnant women are at risk, there has been no formal evaluation of PsA-TT vaccine in pregnant women. The Global Advisory Committee on Vaccine Safety (GACVS) of the World Health Organization (WHO) recommended such an evaluation. This recommendation was the outcome of a meeting held in December 2011, where the Strategic Advisory Group of Experts asked GACVS to provide support to the review of current evidence on the safety of vaccinations in pregnant and lactating women [5]. At this time, Ghana was one of the next countries that had still to implement widespread immunization using this vaccine, and the country also had a demographic and epidemiologic surveillance site in the northern sector (in the meningitis belt), thus providing a unique opportunity to obtain this assessment.

The Navrongo Health Research Centre (NHRC) is located in the Kassena-Nankana municipality in the Upper East Region in northeastern Ghana and is 1 of the 3 field research centers of the Ghana Health Service with a specific mandate to research prevailing health issues in the 3 northern regions of Ghana. Geographically, the Navrongo Health and Demographic Surveillance area lies between latitude 10°30′N and 11°00′N and longitude 1°30′W and covers a total land area of about 1675 km^2^. The estimated population of the surveillance area is about 157 000 living in about 30 000 households, all of whom are under continuous demographic surveillance [6]. Within this area, there is 1 district hospital, 7 health centers, and 27 community health compounds as well as other primary care clinics.

Since 1993, the NHRC has maintained a Health and Demographic Surveillance System, a longitudinal population registration system that monitors the health and demographic dynamics of the study area. Vital demographic events such as pregnancies, births, vaccination, marriages, deaths, and migration are updated every 4 months. Sociodemographic characteristics are collected annually. Verbal autopsies are conducted to elucidate the circumstances surrounding and possible causes of all recorded deaths. To enhance spatial analysis of those data being collected, the HDSS also runs the data linkage system that enables it to link health facility–based information with vital events. Routine HDSS data collection is performed by field workers who receive initial training for a period of up to 1 month and regular retraining for a period of 1 week between data collection rounds. The NHRC is a member of the International Network for the Demographic Evaluation of Populations and Their Health in Developing Countries (INDEPTH), a global network of members who conduct longitudinal health and demographic evaluation of populations in low- and middle-income countries [7].

The study population was composed of 3 cohorts of women: women who received PsA-TT during the national campaign in Ghana in October 2012, and 2 control groups (women who were pregnant during the vaccination campaign but did not receive vaccine, and a second control group of unvaccinated women who were pregnant during the same time period in the prior year). This study was approved by the ethics review committee of the NHRC and was granted a waiver of review by the WHO ethics review committee.

## METHODS

This was an observational comparative cohort study in which the rates of prespecified events were compared between pregnant women who received PsA-TT and the 2 control groups defined above. Prespecified outcomes included spontaneous abortions (fetal deaths occurring at <28 weeks’ gestation), stillbirths (fetal deaths ≥28 weeks’ gestation), perinatal deaths (infant deaths within 48 hours of the onset of labor), premature birth (<36 weeks’ gestation), very premature birth (<28 weeks’ gestation), low birth weight (<2500 g), small for gestational age, cesarean delivery, and maternal all-cause mortality. All events and vaccine exposure information were routinely identified from the routine scripted demographic surveillance surveys conducted within the Navrongo surveillance population. Gestational age was calculated based on the date of the last menstrual period of the woman and her delivery date. Rates of the events were compared using Poisson regression analyses that adjusted for age and place of residence (rural/urban).

## RESULTS

Overall, a total of 1730 pregnant women were immunized with PsA-TT within the Navrongo surveillance site during the immunization program. Of these, 619 were immunized in the first trimester, 534 in the second trimester, and 577 in the third trimester. The age distribution of the immunized cohort, unimmunized concurrent controls, and the unimmunized historical control groups are shown in Table 1. Information on the trimester of immunization stratified by age is shown in Table 2.

**Table 1.** Age Distribution of the 3 Cohorts of Pregnant Women in Navrongo

Age Group, y	Immunized Cohort 2012	Unimmunized Historical Controls	Unimmunized Concurrent Controls 2012
15–19	276	562	114
20–24	463	950	191
25–29	431	880	170
30–34	330	676	217
35–39	153	320	135
40–44	60	127	68
45–49	17	36	24
Total	1730	3551	919

**Table 2.** Trimester of Immunization for the Immunized Cohort, Stratified by Age

Age Group, y	Trimester			
	First	Second	Third	Total
15–19	106	68	102	276
20–24	165	139	159	463
25–29	172	128	131	431
Total	443	335	392	1170

The mean birth weight and gestational age in the 3 cohorts were 2939 g and 38.0 weeks, 2935 g and 38.0 weeks, and 2896 g and 38.1 weeks in the vaccinated, concurrent control, and historical control groups, respectively. A comparison of 3 different birth weight strata in the vaccinated women with the 2 control groups is shown in Table 3. There were no significant differences in the birth weight strata distributions between the 3 groups.

**Table 3.** Comparison of Birth Weights in Vaccinated Women Versus Controls

Outcome	Group A Cohort (n = 1730)	Group A Rate/100	Concurrent Control Cohort (n = 921)	Control Rate/100	IRR	95% CI	*P* Value
Birth weight (>2500 g)	1591	92.0	843	91.5			
Low birth weight (<2500 g)	132	7.6	70	7.6	0.98	.82–1.17	.82
Very low birth weight (<1500 g)	7	0.4	6	0.7	0.85	.40–1.78	.66

Outcome	Group A Cohort (n = 1730)	Group A Rate/100	Historical Control Cohort (n = 3551)	Control Rate/100	IRR	95% CI	*P* Value
Birth weight (>2500 g)	1591	92.0	3219	90.7			
Low birth weight (<2500 g)	132	7.6	309	8.7	0.90	.75–1.08	.25
Very low birth weight (<1500 g)	7	0.4	23	0.6	0.78	.39–1.56	.49

Abbreviations: CI, confidence interval; IRR, incidence rate ratio.

Similarly, when birth outcomes and delivery mode were also compared between vaccinated women and the 2 control groups, no significant differences were observed, as can be seen in Table 4. Of note is that data on delivery method were not available for the historical control group; hence, such comparisons were not possible. In Table 5, miscarriages were further evaluated to look for clustering of this event following vaccination. No such clustering was seen (*P* = .25). Similarly, for stillbirth there was no clustering in time between the date of vaccination and the event (*P* = .44). Comparisons for neonatal outcomes are shown in Table 6. There were no significant differences in the rates of prematurity or neonatal mortality between vaccine recipients and controls.

**Table 4.** Comparison of Birth Outcome and Delivery Mode Between Vaccinated Pregnant Women and Controls

Outcome	Group A Cohort (n = 1730)	Group A Rate/100	Concurrent Controls (n = 919)	Control Rate/100	IRR	95% CI	*P* Value
Live birth	1692	97.8	899	97.8			
Stillbirth	22	1.3	14	1.5	0.95	.62–1.46	.80
Miscarriage	16	0.9	6	0.7	1.06	.65–1.74	.82
Maternal Mortality	0	0	3	0.3	…	…	…
Normal delivery	1642	94.9	871	94.8			
Cesarean delivery	37	2.1	23	2.5	0.95	.69–1.32	.77
Vacuum delivery	8	0.5	7	0.8	0.82	.41–1.63	

Outcome	Group A Cohort (n = 1730)	Group A Rate/100	Historical Controls (n = 3551)	Control Rate/100	IRR	95% CI	*P* Value
Live birth	1692	97.8	3468	97.7			
Still birth	22	1.3	47	1.3	0.97	.64–1.48	.89
Miscarriage	16	0.9	36	1.0	0.94	.58–1.54	.80
Maternal Mortality	0	0	0	0	…	…	…

Abbreviations: CI, confidence interval; IRR, incidence rate ratio.

**Table 5.** Timing of Miscarriages Observed in the Vaccine Group

Interval Between Vaccination and Event, wk	No. (%)
1	2 (9.1)
3	5 (22.8)
4	1 (4.5)
5	1 (4.5)
8	2 (9.1)
12	5 (22.8)
17	6 (27.2)
Total	22 cases

**Table 6.** Neonatal Outcomes

Outcome	Group A Cohort (n = 1730)	Group A Rate/100	Control Cohort	Control Rate/100	IRR	95% CI	*P* Value
Concurrent controls (n = 921)							
Prematurity	62	3.6	29	3.1	1.0	.81–1.34	.76
Neonatal mortality	12	0.7	9	1.0	0.88	.50–1.54	.65
Historical controls (n = 3551)							
Prematurity	62	3.6	197	5.6	0.72	.56–.93	.011
Neonatal mortality	12	0.7	42	1.2	0.68	.38–1.19	.179

Abbreviations: CI, confidence interval; IRR, incidence rate ratio.

## DISCUSSION

To the best of our knowledge, this is the first evaluation of the group A meningococcal conjugate vaccine, PsA-TT, in pregnant women. Although the number of women in this study is not extremely large, the results in >1700 pregnant women show no evidence of any safety concerns for the multiple maternal and neonatal outcomes measured. Given that pregnant women in the African meningitis belt and their infants are at high risk of disease sequelae and death due to group A meningococcal disease, these results should provide support for the current recommendation to vaccinate pregnant women in this area with this vaccine.

The results of this study also have important implications for the feasibility of conducting large phase 4 assessments of vaccine safety in developing countries. By employing and building on existing epidemiologic infrastructure and expertise within the INDEPTH network at the Navrongo site that has been developed for other purposes, it has been possible to perform a thorough assessment of this vaccine. This has important implications for future vaccine introductions where such infrastructure at INDEPTH sites and other sites with similar infrastructure could be employed to assure that newly introduced vaccines are safe and that public confidence of vaccination programs is maintained in low- and middle-income countries introducing the vaccine.

In conclusion, it has been possible to conduct a comprehensive evaluation of the PsA-TT vaccine at the Navrongo epidemiologic surveillance site in Ghana. This evaluation has revealed no evidence of any safety concerns when this vaccine was used in pregnant women. Although this study was performed within Ghana alone, and the background rates of events might vary from country to country, the results and conclusions of this study are generalizable to other countries in the African meningitis belt that have similar demographic characteristics.
